# Supplementary material for: Identifying and tailoring C–N coupling site for efficient urea synthesis over diatomic Fe–Ni catalyst
Source: Nat Commun. 2022 Sep 10;13:5337. doi: 10.1038/s41467-022-33066-6 (PMC9464195; doi:10.1038/s41467-022-33066-6)
Supplement: Supplementary file 1 — Supplementary Information [file 41467_2022_33066_MOESM1_ESM.pdf]

Supplementary Information

Identifying and tailoring C-N coupling site for efficient urea synthesis over diatomic Fe-Ni catalyst

Xiaoran Zhang[1,2,‡], Xiaorong Zhu[3,‡], Shuowen Bo[4,‡], Chen Chen[1,*], Mengyi Qiu[1], Xiaoxiao Wei[1], Nihan He[1], Chao Xie[1], Wei Chen[1], Jianyun Zheng[1,*], Pinsong Chen[2], San Ping Jiang[2,5*], Yafei Li[3], Qinghua Liu[4] and Shuangyin Wang[1,*]

[1]State Key Laboratory of Chem/Bio-Sensing and Chemometrics, College of Chemistry and Chemical Engineering, Hunan University, Changsha, P. R. China.

[2]WA School of Mines: Minerals, Energy & Chemical Engineering, Curtin University, Perth, Western Australia 6102, Australia.

[3]College of Chemistry and Materials Science, Nanjing Normal University, Nanjing, Jiangsu, P. R. China.

[4]National Synchrotron Radiation Laboratory, University of Science and Technology of China, Hefei, P. R. China.

[5]Foshan Xianhu Laboratory of the Advanced Energy Science and Technology Guangdong Laboratory, Foshan 528216, China.

[‡]There authors contributed equally to this work.

E-mail: chenc@hnu.edu.cn; jyzheng@hnu.edu.cn; s.jiang@curtin.edu.au; shuangyinwang@hnu.edu.cn

Table of contents

Supplementary Figures 1-34

Supplementary Tables 1-2

Supplementary References 1-4

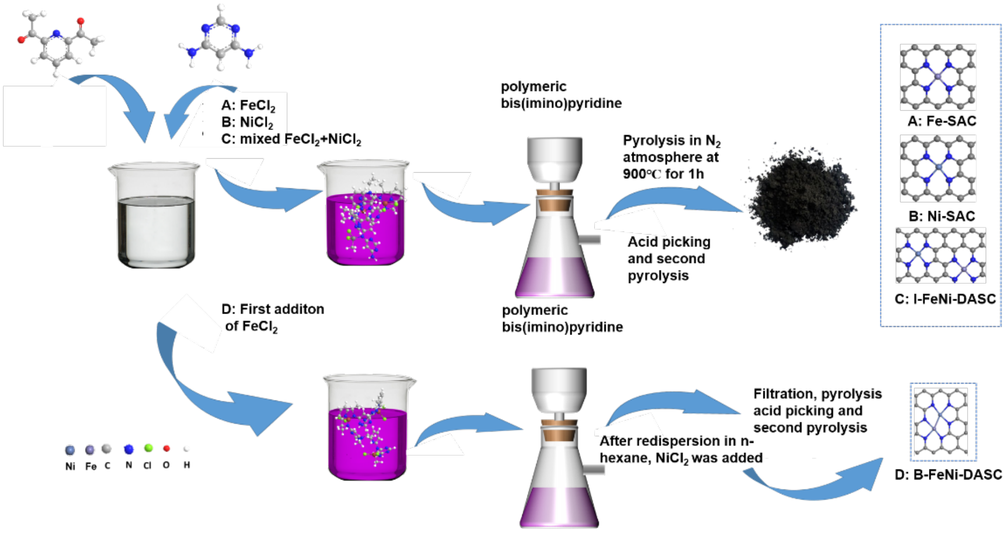

Supplementary Figure 1 | Schematic illustration of the synthesis of Fe-SAC, Ni-SAC, I-FeNi-DASC and B-FeNi-DASC.

A series of single-atom catalysts (Fe-SAC and Ni-SAC), isolated diatomic catalyst (I-FeNi-SAC) and diatomic electrocatalyst with bonded Fe-Ni pair sites (B-FeNi-DASC) decorated on nitrogen-doped carbon support were obtained via pyrolysis of the coordination polymer (Bis(imino)-pyridyl ligands) as illustrated in Supplementary Fig. 1, which derived from Schiff base condensation reaction with 2,4-diaminopyrimidine and 2,6-diacetylpyridine as reactants[1].

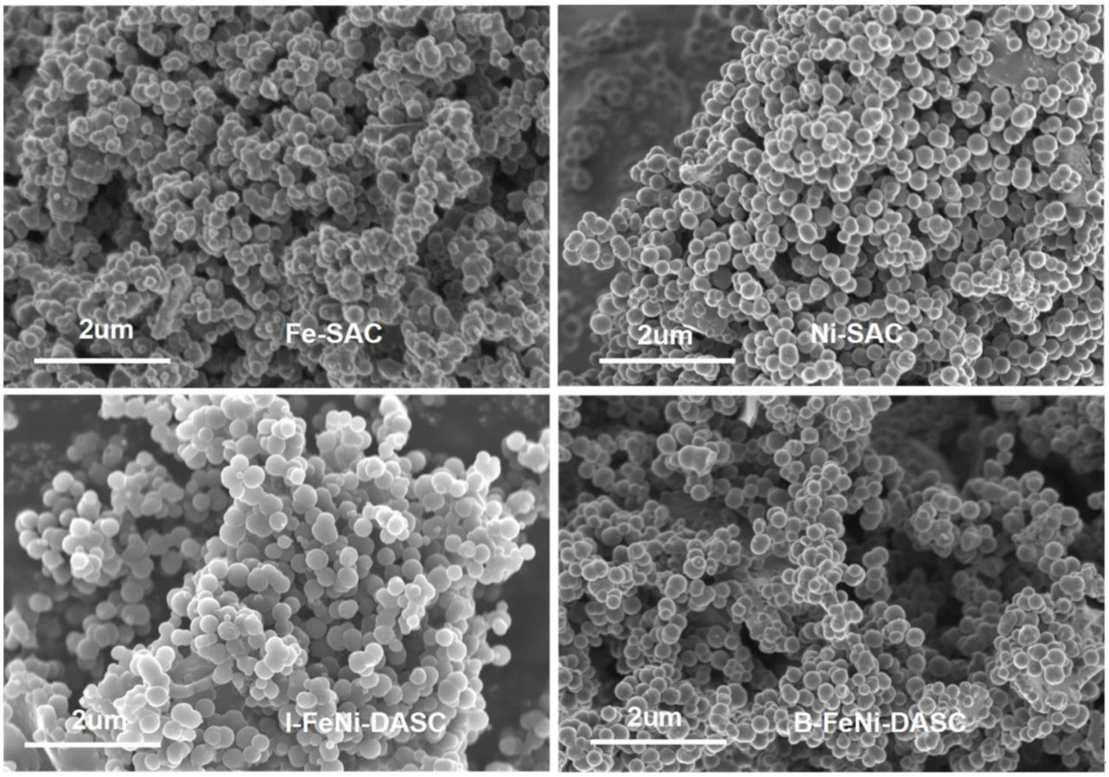

Supplementary Figure 2 | SEM images of Fe-SAC, Ni-SAC, I-FeNi-DASC and B-FeNi-DASC.

As shown in Supplementary Fig. 2, those catalysts display uniform nanosphere microstructure which derived from the self-assembly and polymerization of compounds containing amino and acyl groups.

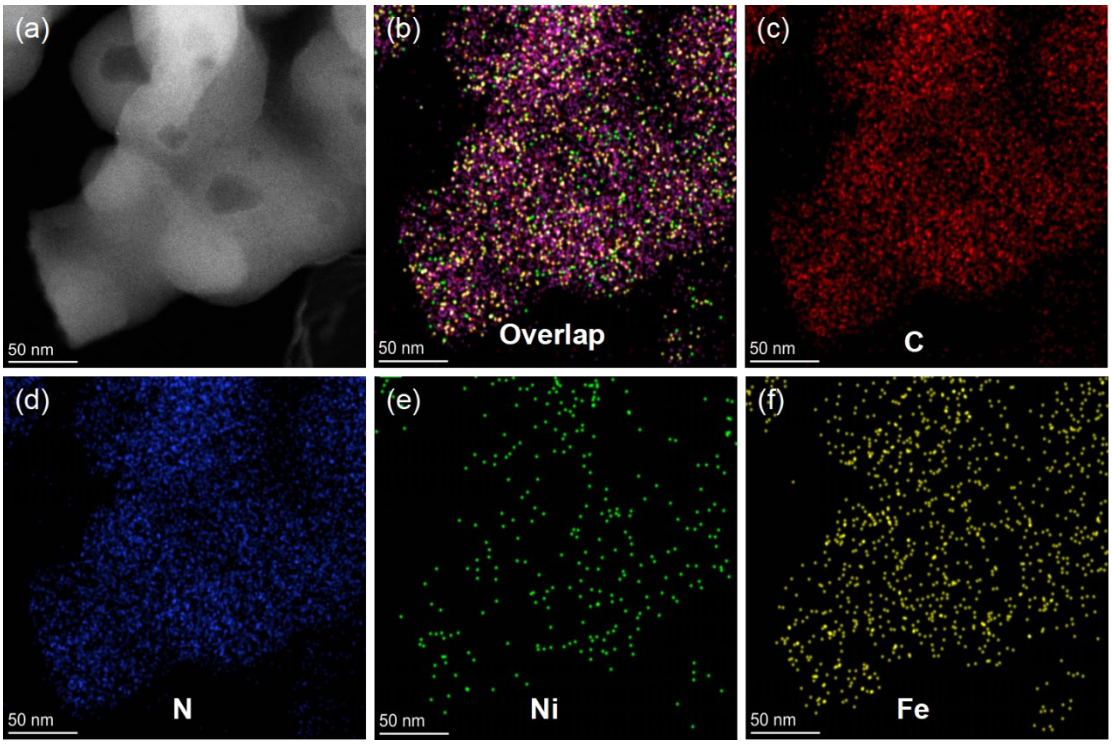

Supplementary Figure 3 | The elemental mapping images of B-FeNi-DASC.

The uniform dispersion of C, N, Fe and Ni elements over the carbon matrix was supported by the elemental mapping of the B-FeNi-DASC as illustrated in Supplementary Fig. 3.

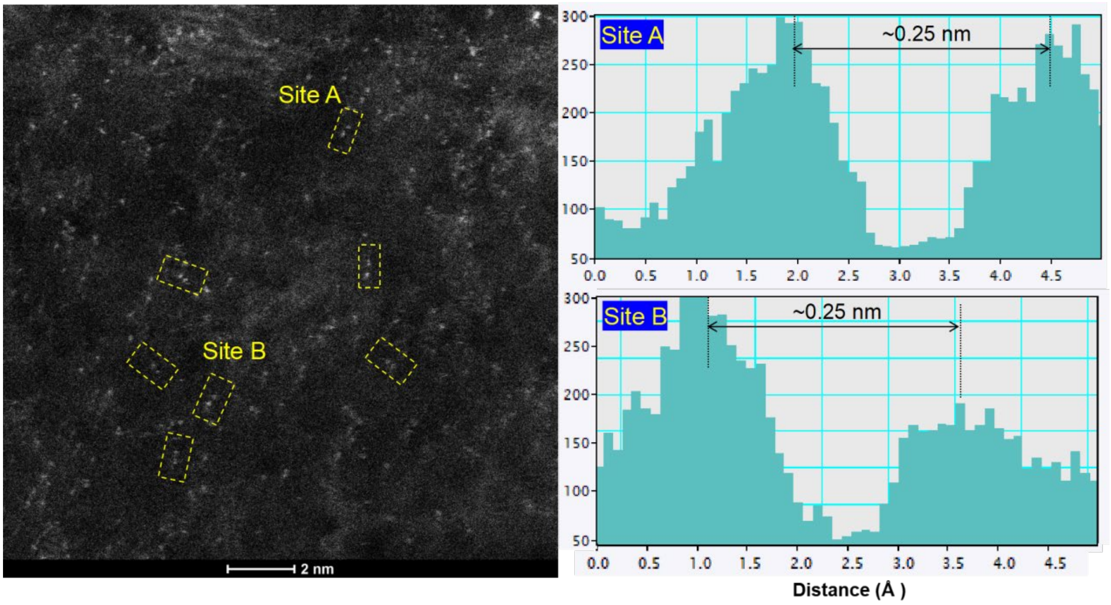

Supplementary Figure 4 | Electron energy-loss spectroscopy (EELS) line scan of atomic pairs.

The atomic pair of Fe-Ni exhibits the typical distance of about 0.25 nm, serving as the solid evidence for the successful synthesis of bonded Fe-Ni site configurations, as indicated in Supplementary Figure 4.

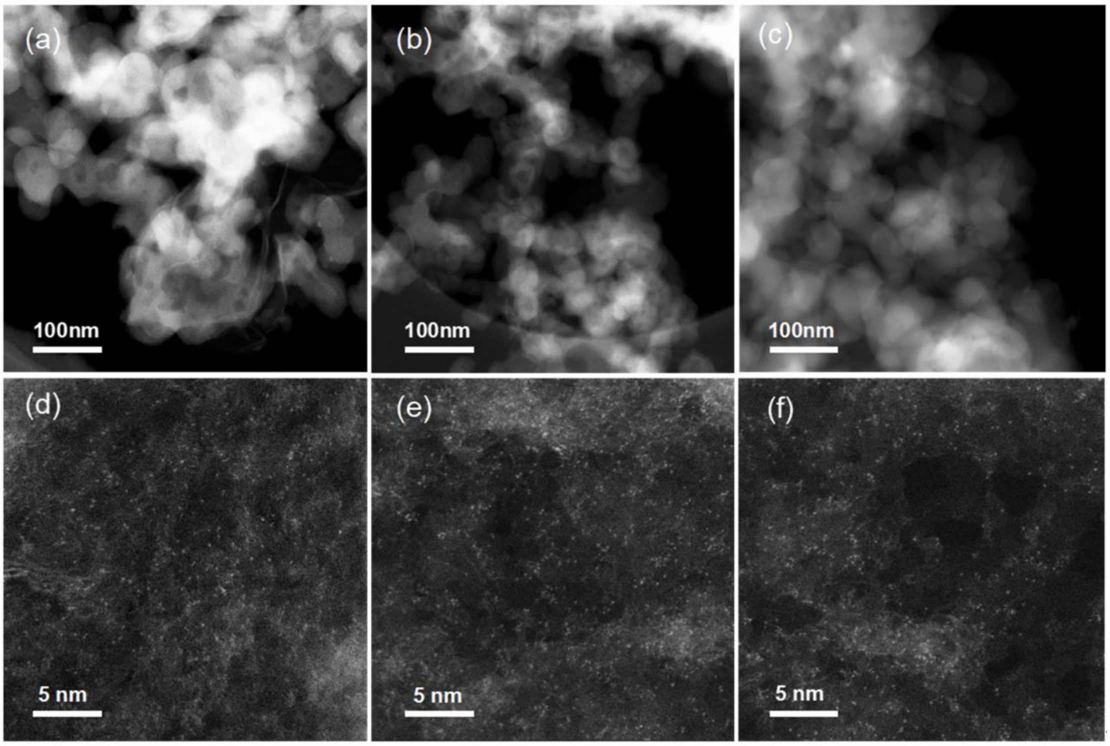

Supplementary Figure 5 | STEM images of (a, d) Fe-SAC. (b, e) Ni-SAC. (c, f) I-FeNi-DASC.

The STEM images of the above electrocatalysts show that there are highly dispersed bright dots anchored on carbon matrix, and there are no obvious metallic nanoparticles and bright dots aggregation appeared in Fe-SAC, Ni-SAC and I-FeNi-DASC, demonstrating that iron and nickel sites are exist with the form of single atoms in those materials.

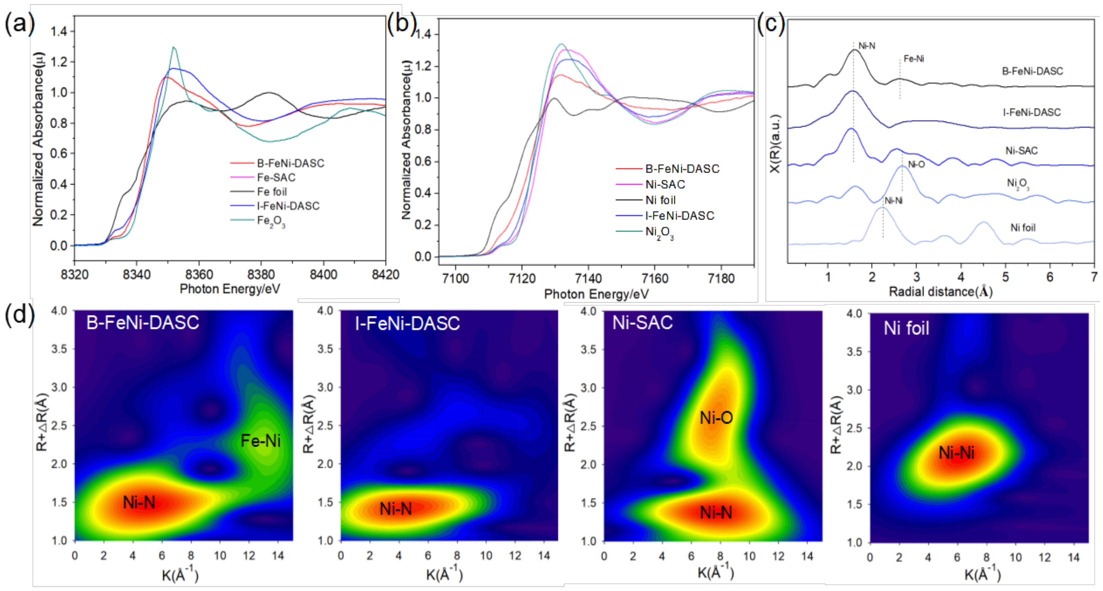

Supplementary Figure 6 | Normalized (a) Ni, (b) Fe *K*-edge XANES spectra of Ni foil, Fe, Fe_2O_3, Ni_2O_3, Fe-SAC, Ni-SAC, I-FeNi-DASC and B-FeNi-DASC. (c) Experimental Fourier transform at the Ni *K*-edge of EXAFS data of Ni foil, Fe, Fe_2O_3, Ni_2O_3, Fe-SAC, Ni-SAC, I-FeNi-DASC and B-FeNi-DASC. (d) Wavelet transforms for EXAFS signals of Fe-SAC, Ni-SAC, I-FeNi-DASC and B-FeNi-DASC.

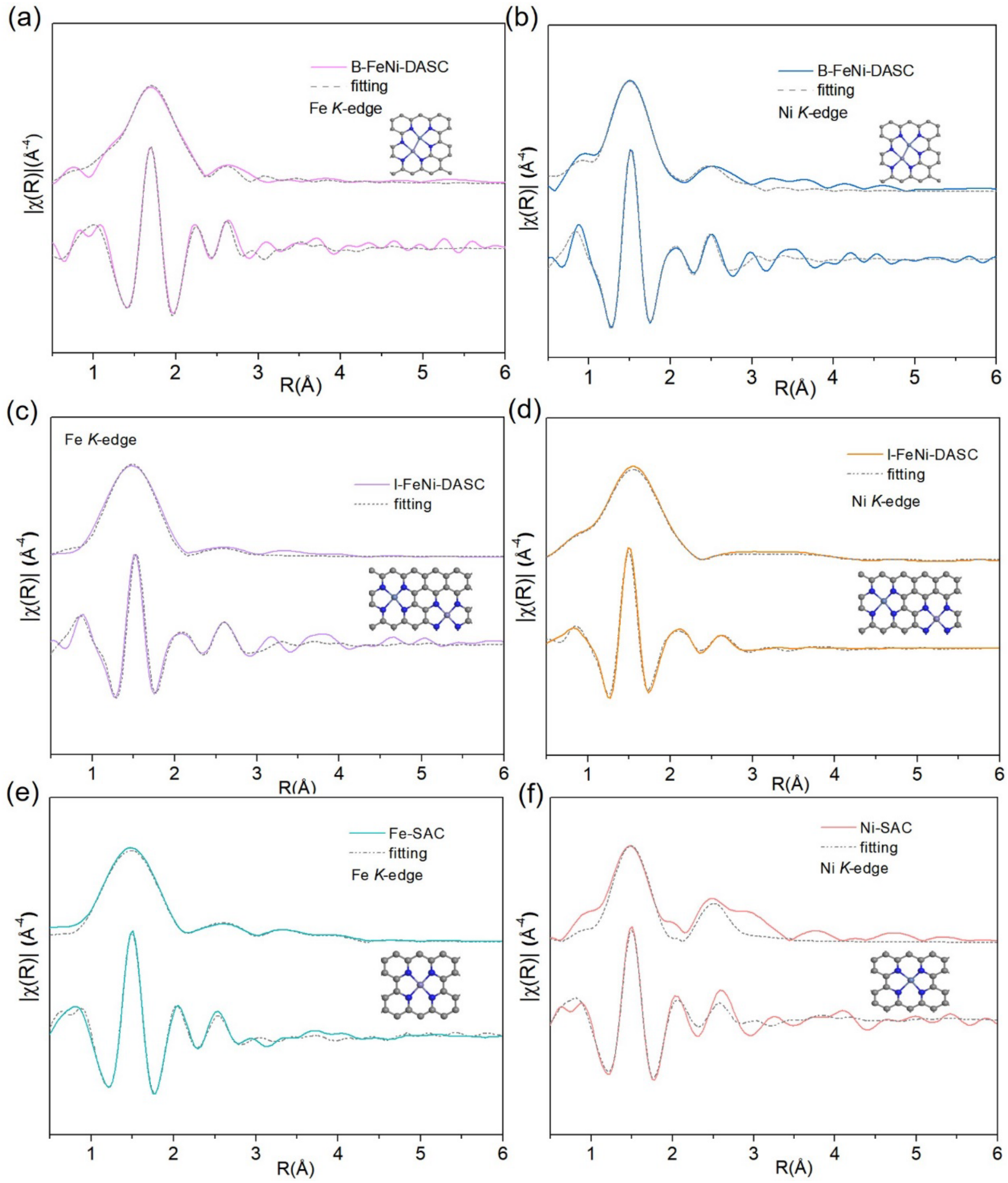

Supplementary Figure 7 | Experimental and fitted EXAFS spectra of the Fe *K*-edge in (a) B-FeNi-DASC, (b) Ni *K*-edge in B-FeNi-DASC, (c) Fe *K*-edge in I-FeNi-DASC, (d) Ni *K*-edge in I-FeNi-DASC, (e) Fe *K*-edge in Fe-SAC and (f) Ni *K*-edge in Ni-SAC. The insets show the corresponding fitted structural modeling. Purple, blue, navy blue and grey spheres refer to Fe, Ni, N and C atoms, respectively.

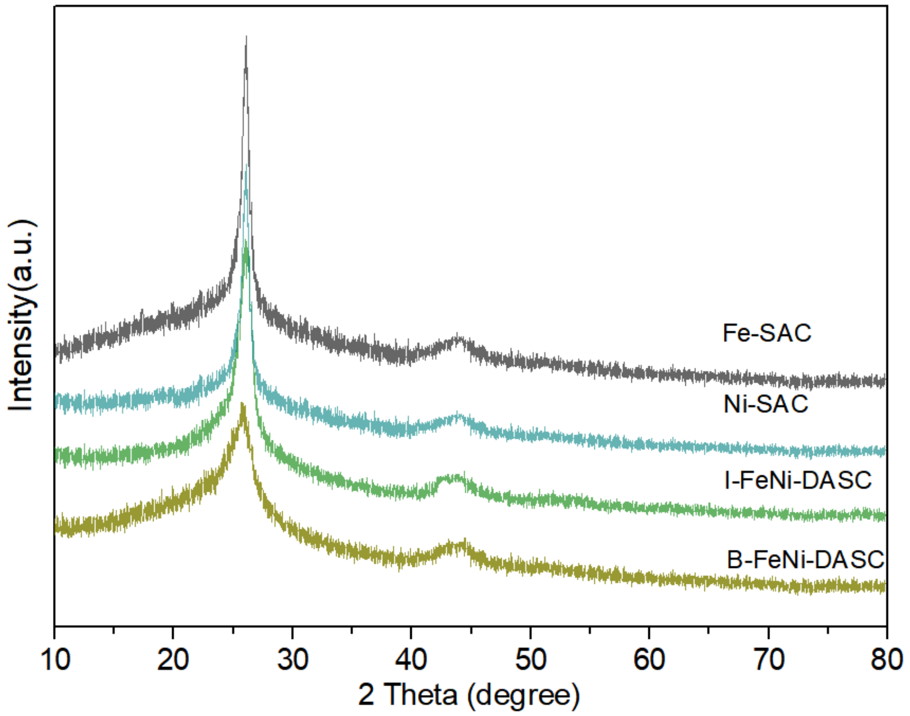

Supplementary Figure 8 | X-ray diffraction (XRD) spectra of Fe-SAC, Ni-SAC, I-FeNi-DASC and B-FeNi-DASC.

The crystal structure of the these electrocatalysts were analyzed by X-ray diffraction spectroscopy, which shows that the four catalysts share similar XRD peaks located at around 26.3° associated with (002) planes of graphitic carbon (PDF#41-1487) and weak diffraction peak at around 43.5° overlapped with that (100) plane of graphitized carbon. Meanwhile, there are no peaks associated with the metallic phase, suggesting the successful fabrication of material with atomically dispersed sites[2].

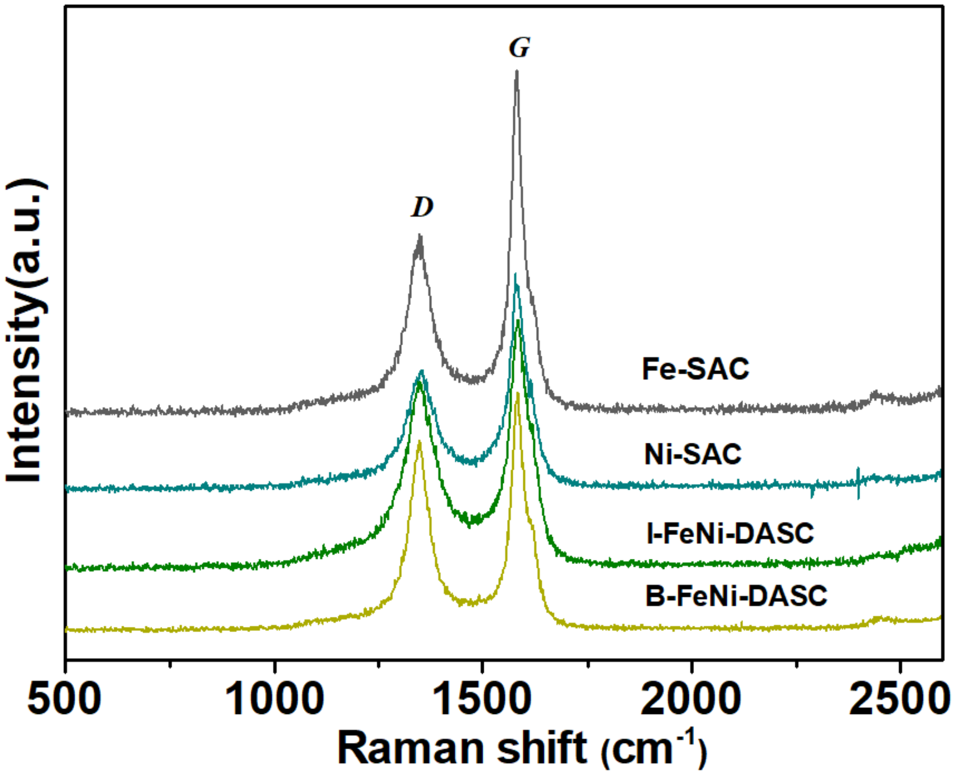

Supplementary Figure 9 | Raman spectra of Fe-SAC, Ni-SAC, I-FeNi-DASC and B-FeNi-DASC.

As presented in Supplementary Fig. 9, Raman spectra indicate that defects exist in those materials, which may be derived from polymer pyrolysis as well as the introduction of Fe, Ni, N, O dopants into carbon matrix. In addition, it is found that these samples share the similar degree of graphitization.

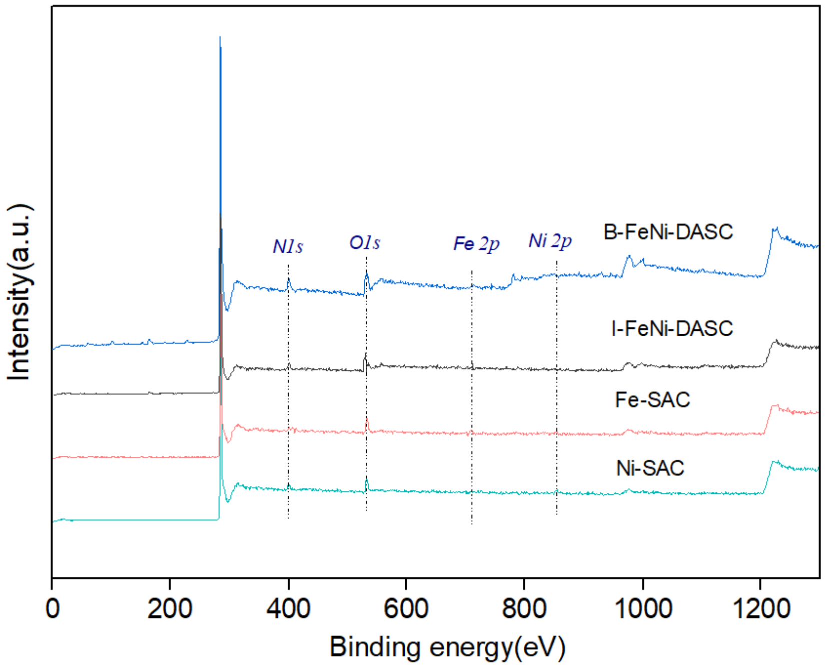

Supplementary Figure 10 | The XPS survey scans in Fe-SAC, Ni-SAC, I-FeNi-DASC and B-FeNi-DASC.

The XPS survey spectra of the single-atom and diatomic electrocatalysts clearly show the presence of corresponding C, N, O and Fe, Ni elements respectively, serving as the evidence for the successful doping of Fe, Ni, or co-doping of Fe and Ni into carbon matrix.

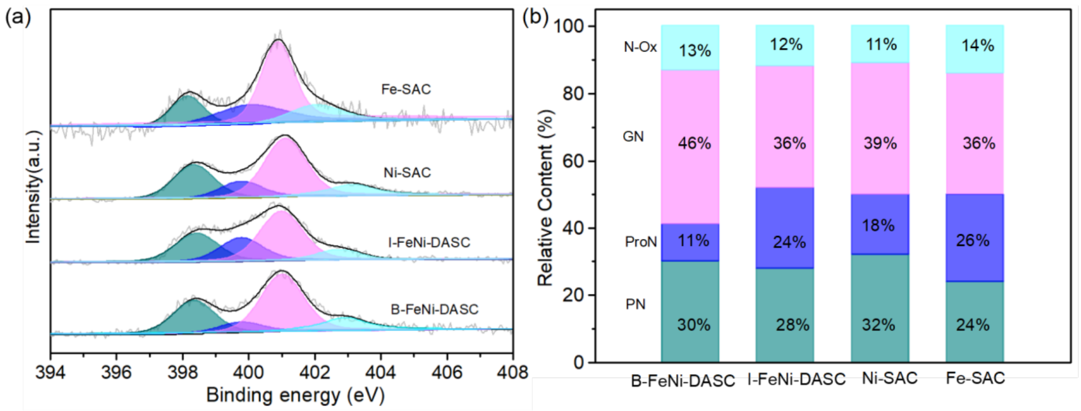

Supplementary Figure 11 | High-resolution XPS spectra of N *1s* in Fe-SAC, Ni-SAC, I-FeNi-DASC and B-FeNi-DASC.

Nitrogen is the most important heteroatom dopant in enhancing and manipulating the carbon based electrocatalysts. In single-atom system, the doped N not only acts as the central element of the TM-N_4 center, but also as the host dopant element with high content widely distributed on the carbon matrix around the TM-N_4. The presence of various N species would have significant effect on the chemical and electronic surrounding environment of TM-N_4 sites. The contents of each N species in these samples were detected (Supplementary Fig. 11). The N *1s* spectra were deconvoluted into four peaks of pyridinic N (398.4 eV), graphitic N (399.9 eV), pyrrolic N (401.3 eV) and oxidized N (403.2 eV)[3].

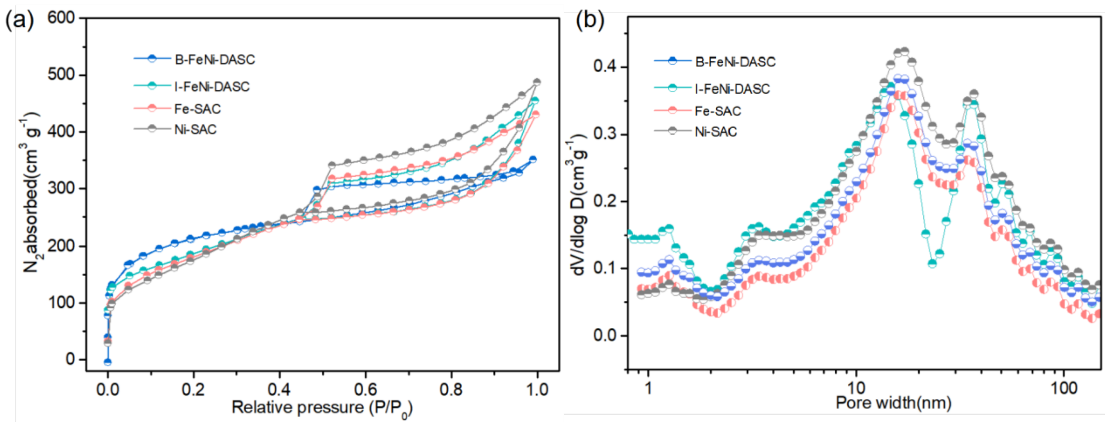

Supplementary Figure 12 | (a) Nitrogen adsorption-desorption isotherms and (b) pore size distribution for Fe-SAC, Ni-SAC, I-FeNi-DASC and B-FeNi-DASC.

Sufficient specific surface area and rational pore structure are conducive to exposing active sites and mass transfer during catalytic process for urea synthesis. Brunauer-Emmett-Teller (BET) surface area and pore size distribution information of these materials are list in Supplementary Fig. 12. It is found that the porous nanospheres anchored with single sites exhibit similar specific surface area of Fe-SAC (864 m^2 g^{-1}), Ni-SAC (872 m^2 g^{-1}), I-FeNi-DASC (907 m^2 g^{-1}) and B-FeNi-DASC (886 m^2 g^{-1}) as well as hierarchical pore structure of partial mesoporous combined with most of micropore, providing a good mass transfer circumstances for catalytic process.

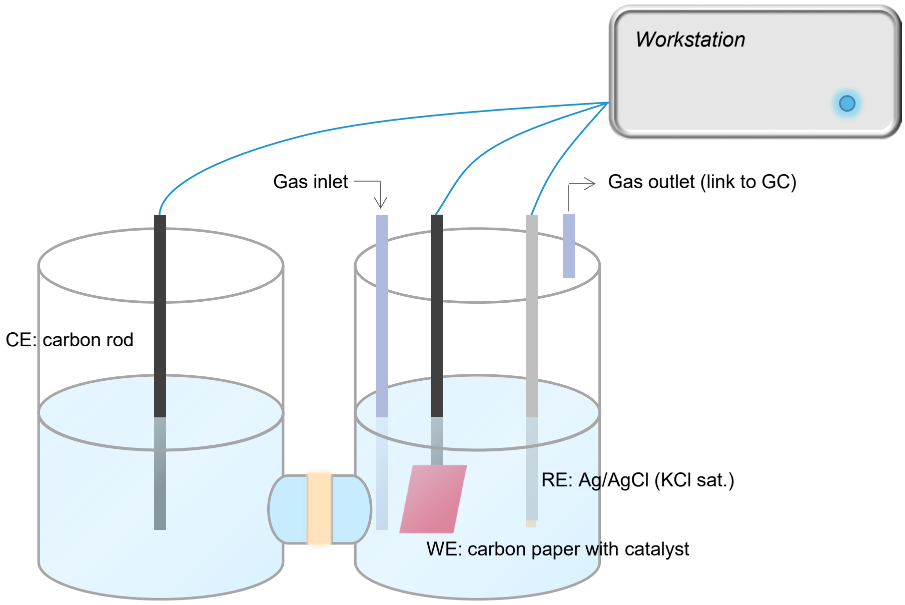

Supplementary Figure 13 | The schematic diagram of electrochemical measuring apparatus.

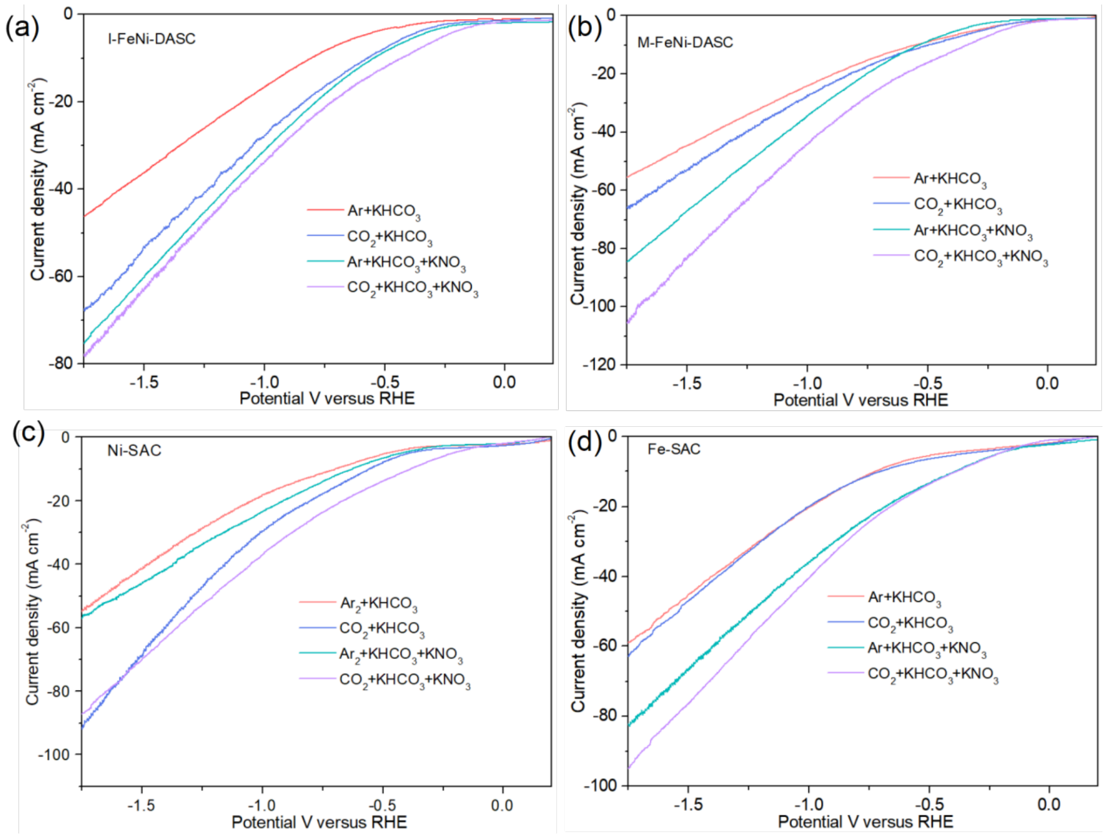

Supplementary Figure 14 | LSV curves for electrolysis of (a) I-FeNi-DASC, (b) M-FeNi-DASC, (c) Ni-SAC and (d) Fe-SAC in the electrolytes of Ar-saturated 0.1 M $KHCO_3$, CO_2-saturated 0.1M$KHCO_3$, Ar-saturated 0.1 M $KHCO_3$ with 50 mM KNO_3 and CO_2-saturated 0.1 M $KHCO_3$ with 50 mM KNO_3 respectively, the scan rate is 10 mV s^{-1}.

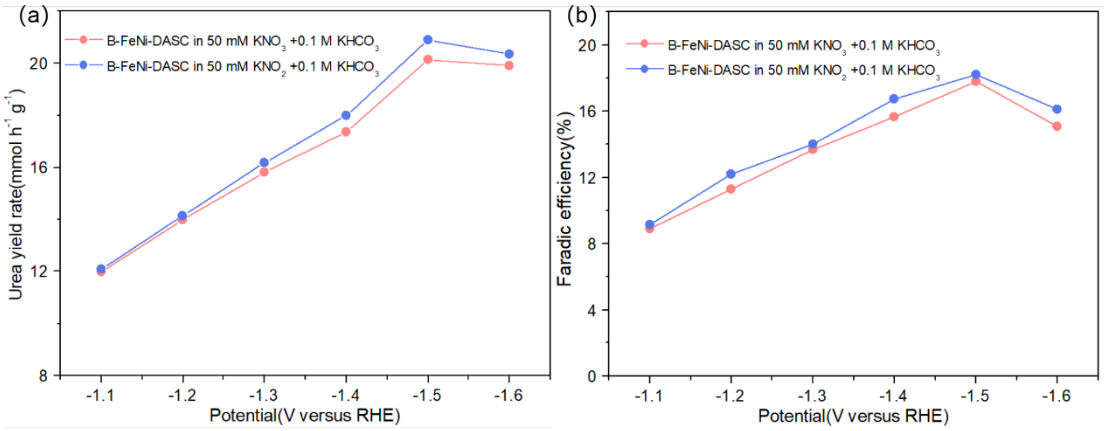

Supplementary Figure 15 | (a) The Urea yield rates and (b) corresponding Faradaic efficiencies of B-FeNi-DASC in electrolytes of CO_2-saturated 0.1 M $KHCO_3$ with 50 mM KNO_3 and CO_2-saturated 0.1 M $KHCO_3$ with 50 mM KNO_2.

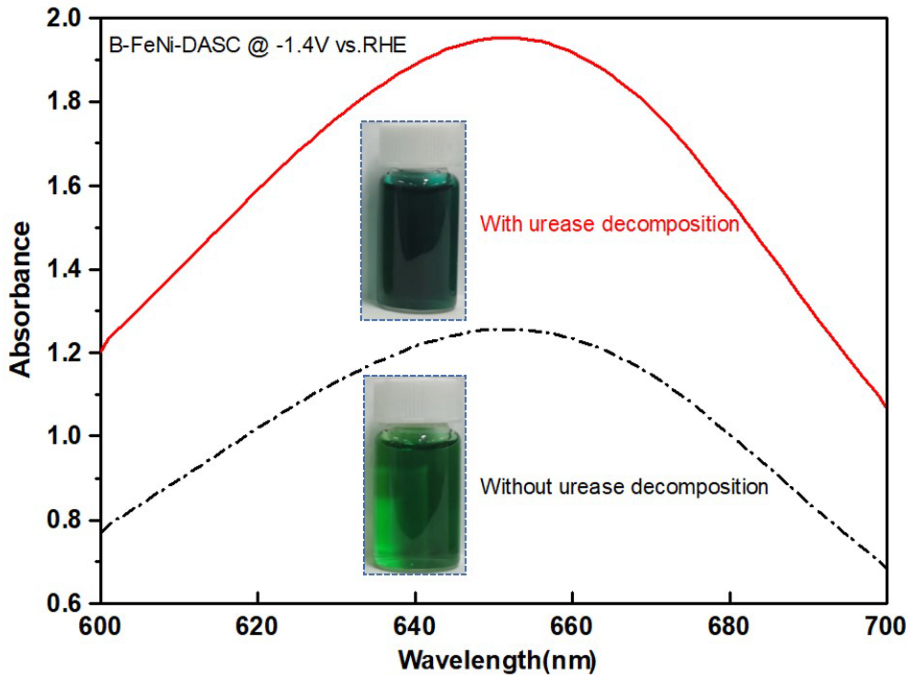

Supplementary Figure 16 | The corresponding absorbance curve by UV-vis spectrophotometer with and without urease decomposition. The absorbance at 662 nm was measured by UV-vis spectrophotometer. The insets are the optical images for color generation assays.

The corresponding absorbance curve by UV-vis spectrophotometer of B-FeNi-DASC under the applied potential of -1.4 V versus RHE with and without urease decomposition were illustrated in Supplementary Figure 16.

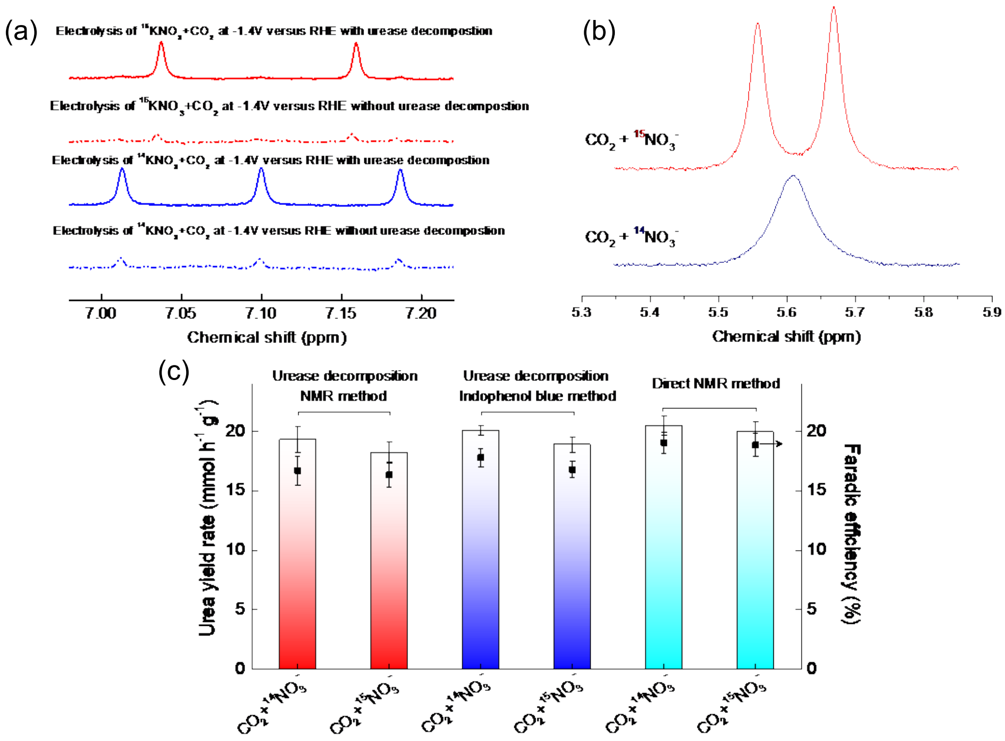

Supplementary Figure 17 | **The identification and quantification for electrochemical urea and ammonia synthesis.** (a) The ^{1}H NMR spectra for urea product-derived ammonia at -1.4 V versus RHE for isotope labeling measurements. (b) The ^{1}H NMR spectra for direct urea product at -1.4 V versus RHE for isotope labeling measurements. (c) The urea yield rates and corresponding Faradaic efficiencies obtained by urease decomposition-NMR method, direct urea NMR method, urease decomposition-indophenol blue method and for isotope labeling measurements at -1.4 V versus RHE. The error bars represent the standard deviation for at least three independent measurements.

As shown in Supplementary Figure 17c, the urea yield rates and Faradaic efficiencies at -1.4 V versus RHE with $^{15}KNO_3$ and $^{14}KNO_3$ as N source show no discernible difference for all of three quantification method, which prove that both urease decomposition-indophenol blue method, urease decomposition-NMR method and the direct NMR method are suitable for urea product quantification. The presented urea yield rates in this work are mainly obtained by the urease decomposition-indophenol blue method. Considering the relatively lower requirement on the NMR instrument (600 MHz instead of 800 MHz) for detection of ammonia, the urease decomposition-NMR method is easier to be realized for qualitative and quantitative analysis of urea products in this field.

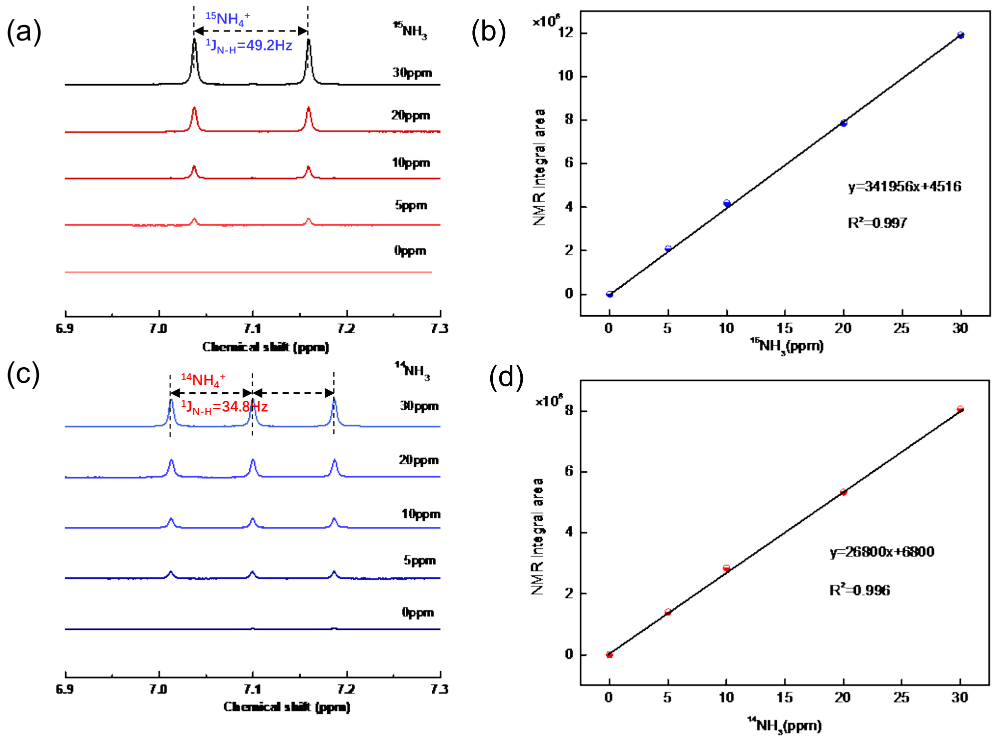

Supplementary Figure 18 | **NMR spectra and calibration curves for quantification of urea-derived ammonia**. (a) NMR spectra of $^{15}NH_4^+$ with various concentrations. (b) The calibration curves for $^{15}NH_3$. (c) NMR spectra of $^{14}NH_4^+$ with various concentrations. (d) The calibration curves for $^{14}NH_3$.

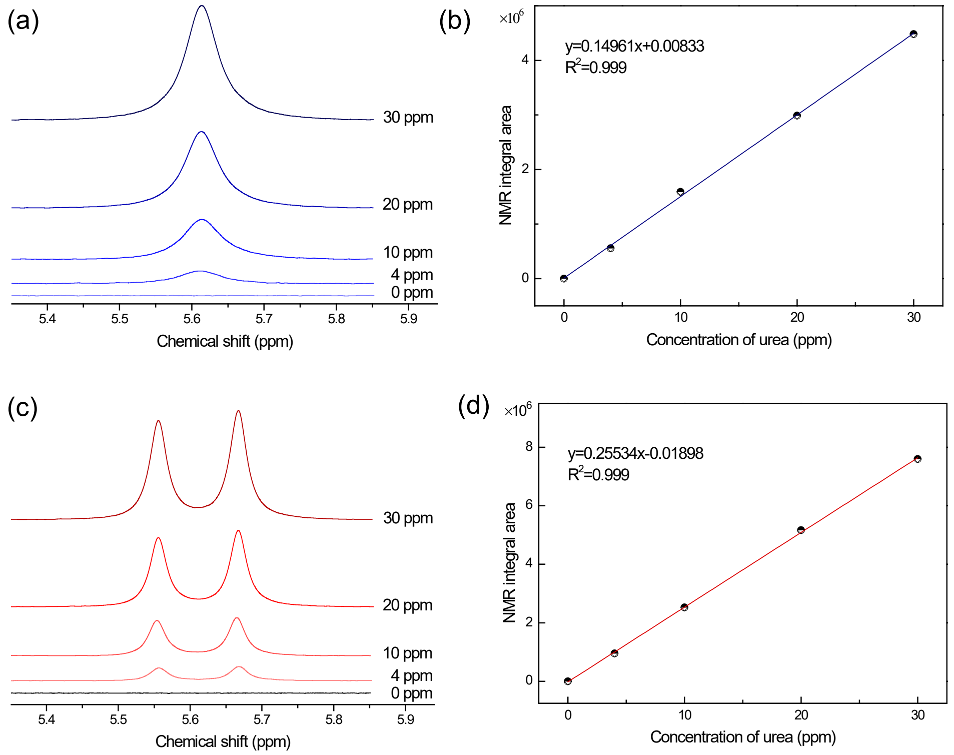

Supplementary Figure 19 | **NMR spectra and calibration curves for quantification of urea**. (a) NMR spectra of $CO(^{14}NH_2)_2$ with various concentrations. (b) The calibration curves for $CO(^{14}NH_2)_2$. (c) NMR spectra of $CO(^{15}NH_2)_2$ with various concentrations. (d) The calibration curves for $CO(^{15}NH_2)_2$.

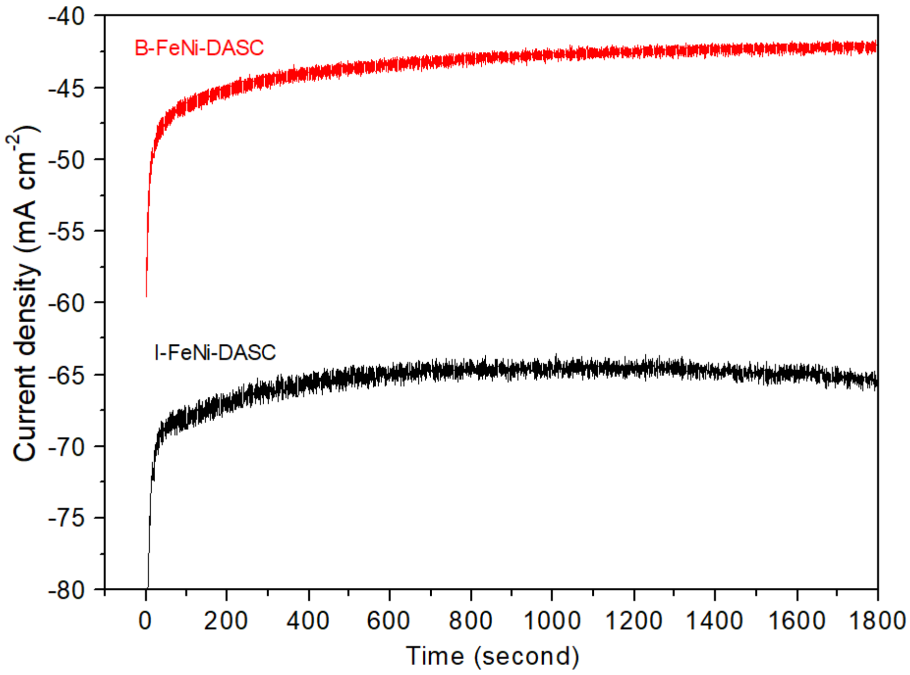

Supplementary Figure 20 | The i-t curves for electrocoupling over B-FeNi-DASC and I-FeNi-DASC at -1.5 V versus RHE.

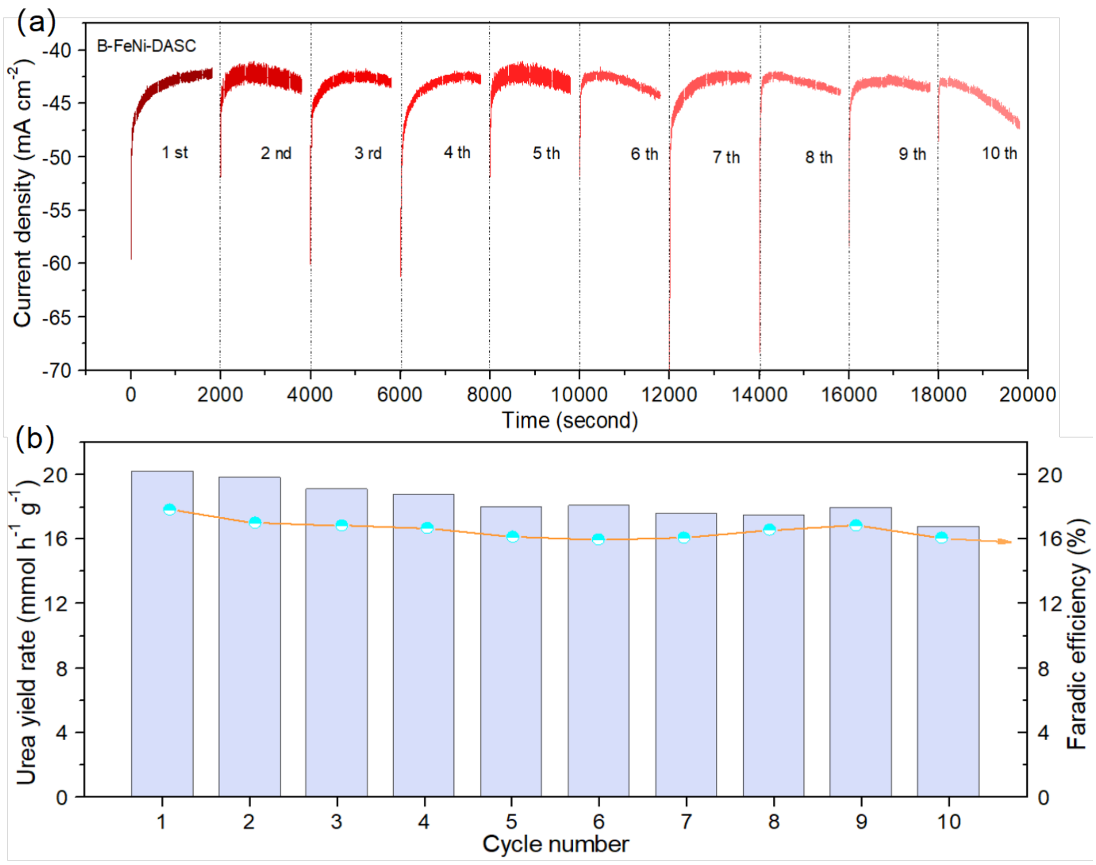

Supplementary Figure 21 | (a) The i-t curves for C-N coupling and (b) urea yield rates and Faradaic efficiencies over B-FeNi-DASC at -1.5 V versus RHE during ten continuous cycles.

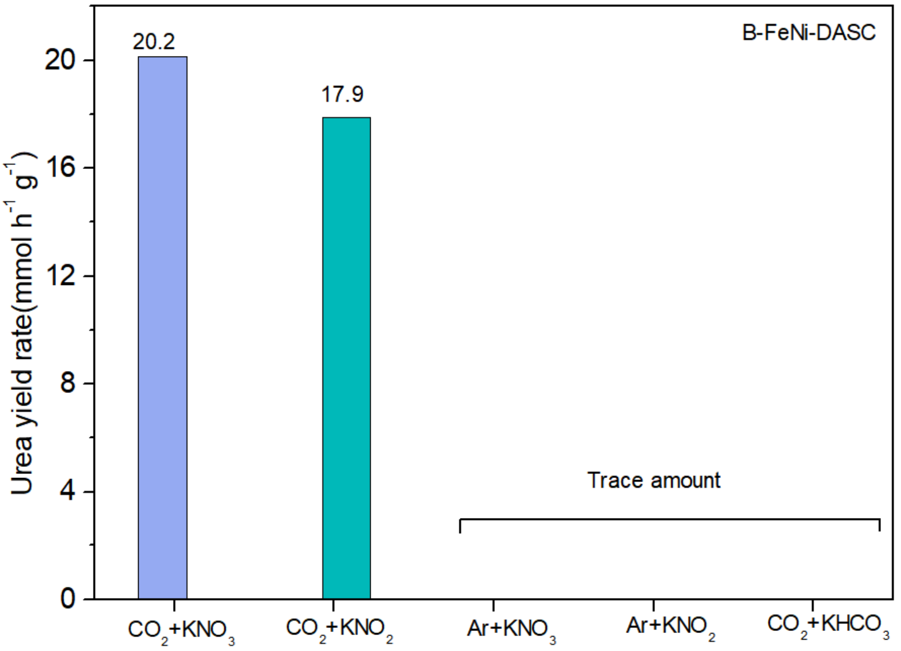

Supplementary Figure 22 | Comparison of urea yield rates in CO_2-saturated 0.1 M $KHCO_3$ with 50 mM KNO_3, CO_2-saturated 0.1 M $KHCO_3$ with 50 mM KNO_2, Ar-saturated 0.1 M $KHCO_3$ with 50 mM KNO_3, Ar-saturated 0.1 M $KHCO_3$ with 50 mM KNO_2 and CO_2-saturated 0.1 M $KHCO_3$, respectively, over B-FeNi-DASC at -1.5 V versus RHE.

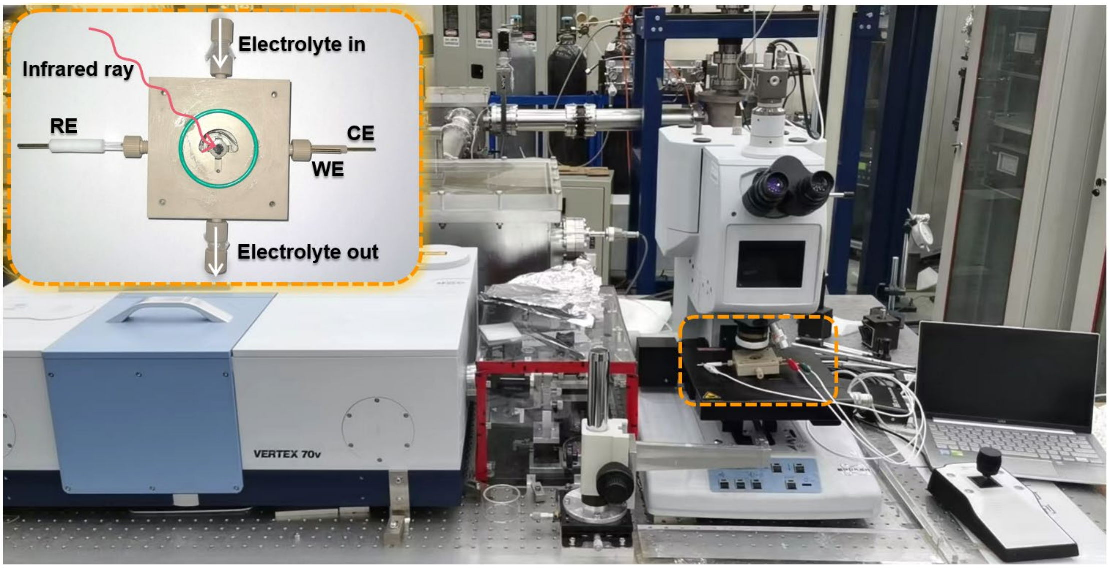

Supplementary Figure 23 | The optical images of instrument for in-situ electrochemical spectroscopy characterization.

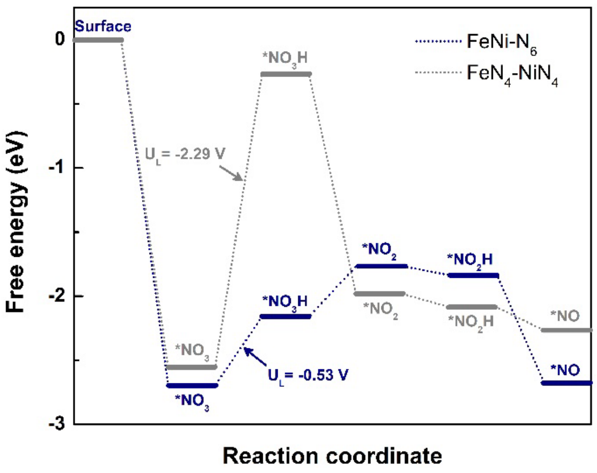

Supplementary Figure 24 | The free energy diagram of NO formation with NO_3^- as the starting point on FeNi-N_6 and FeN_4-NiN_4 slabs.

Infer from the Supplementary Figure 24, we find out that the potential limiting step for NO formation on FeNi-N_6 and FeN_4-NiN_4 is the first hydrogenation process. ($*NO_3 + H^+ + e^- \rightarrow *NO_3H$), corresponding U_L are -0.53 V and -2.29 V for FeNi-N_6 and FeN_4-NiN_4 respectively. The weak $*HNO_3$ adsorption on FeN_4-NiN_4 are too weak to drive the NO formation while the synergy effect between Fe-Ni atoms in FeNi-N_6 greatly enhanced the adsorption strength of $*HNO_3$, making the direct formation of NO feasible.

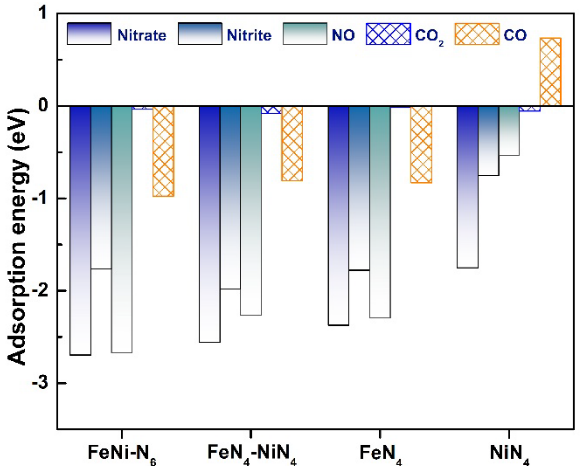

Supplementary Figure 25 | The adsorption energy of nitrate, nitrite, NO, CO_2 and CO on single-atom electrocatalysts (FeN_4, NiN_4) and diatomic electrocatalysts (FeNi-N_6, FeN_4-NiN_4) respectively.

The adsorption energies of CO_2, NO, nitrate, nitrite, and CO over the SACs and isolated/bonded-Fe-Ni-DASCs have been listed in Supplementary Figure 25, supporting the superior urea synthesis performance of FeNi-N_6 pair site.

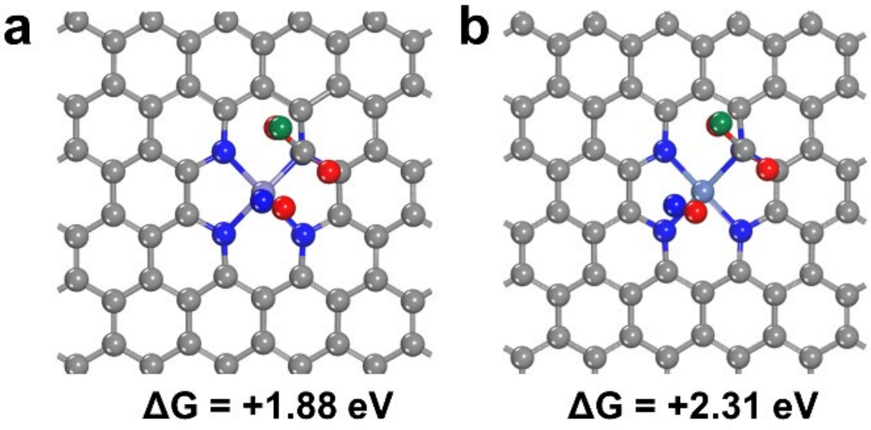

Supplementary Figure 26 | The optimized geometry structures of *NO and *COOH co-adsorbed (a) Fe-Ni_4 and (b) Ni-N_4 respectively. The *COOH formation free energies were also listed.

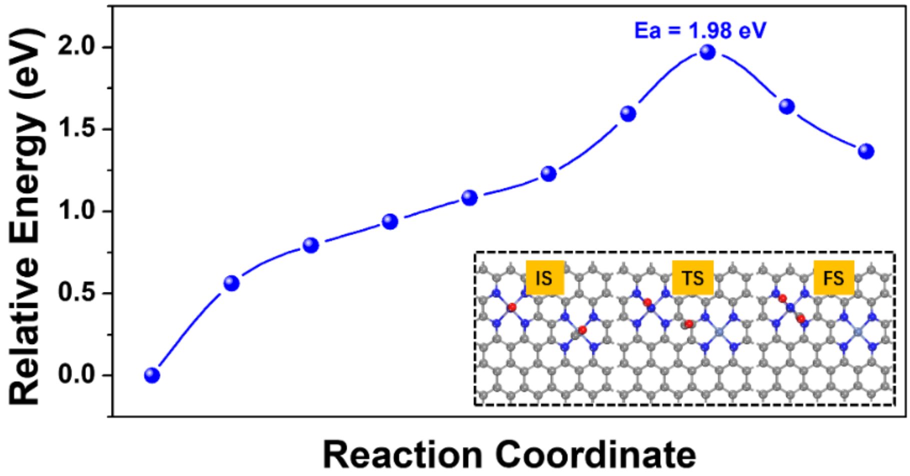

Supplementary Figure 27 | The kinetic energy barrier of *CO+*NO coupling on FeN_4-NiN_4.The insert frame included the optimized structures of initial state, transition state and finial state along the reaction pathway.

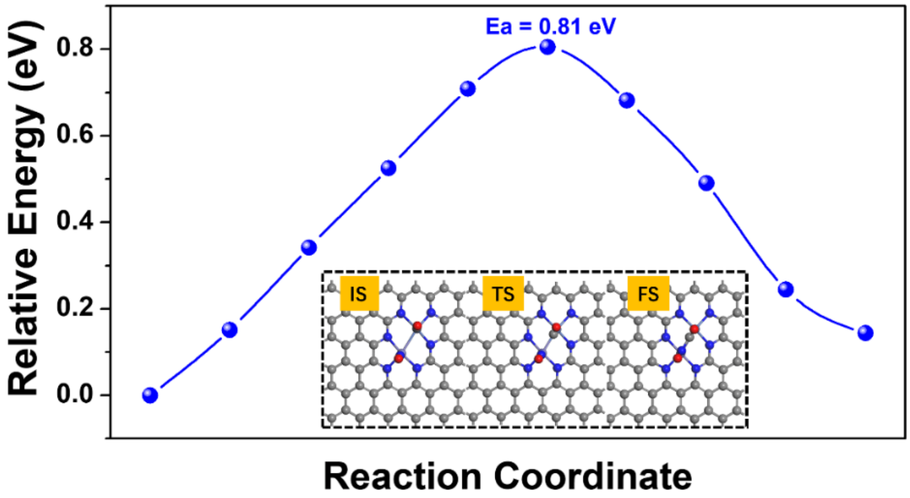

Supplementary Figure 28 | The kinetic energy barrier of *CO+*NO coupling on FeNi-N_6.The insert frame included the optimized structures of initial state, transition state and finial state along the reaction pathway.

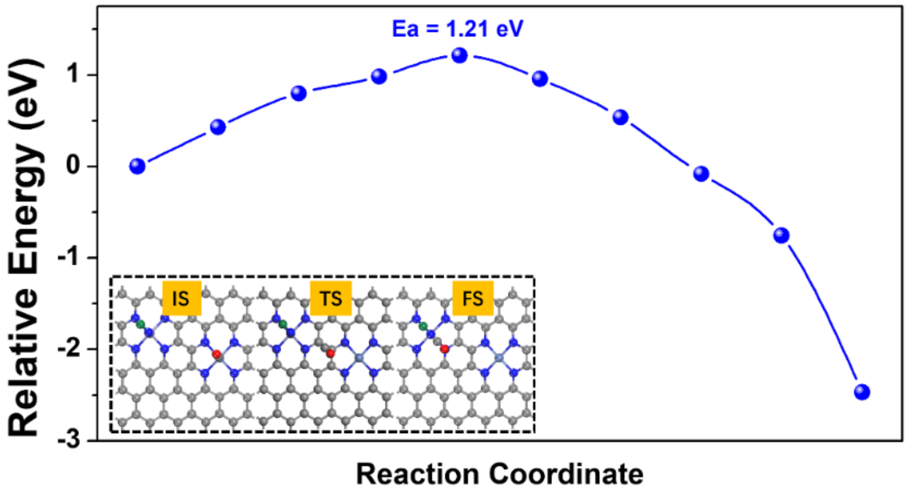

Supplementary Figure 29 | The kinetic energy barrier of *CO+*NH coupling on FeN_4-NiN_4.The insert frame included the optimized structures of initial state, transition state and finial state along the reaction pathway.

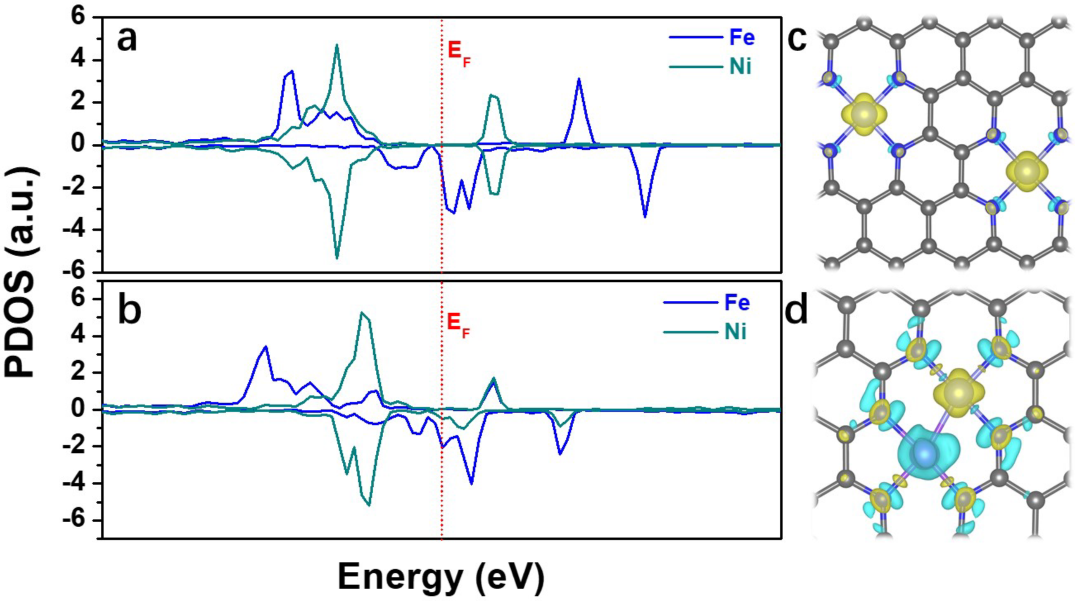

Supplementary Figure 30 | The partial density of states (PDOS) of 3d orbitals of (a) I-FeNi-DASC and (b) B-FeNi-DASC. The differential charge density maps of (c) I-FeNi-DASC and (d) B-FeNi-DASC. The charge density difference with iso surface value of 0.01 e/Bohr^3 of FeNi-N_6 and FeN_4-NiN_4 respectively. Blue and yellow colors represent losing and gaining electrons respectively.

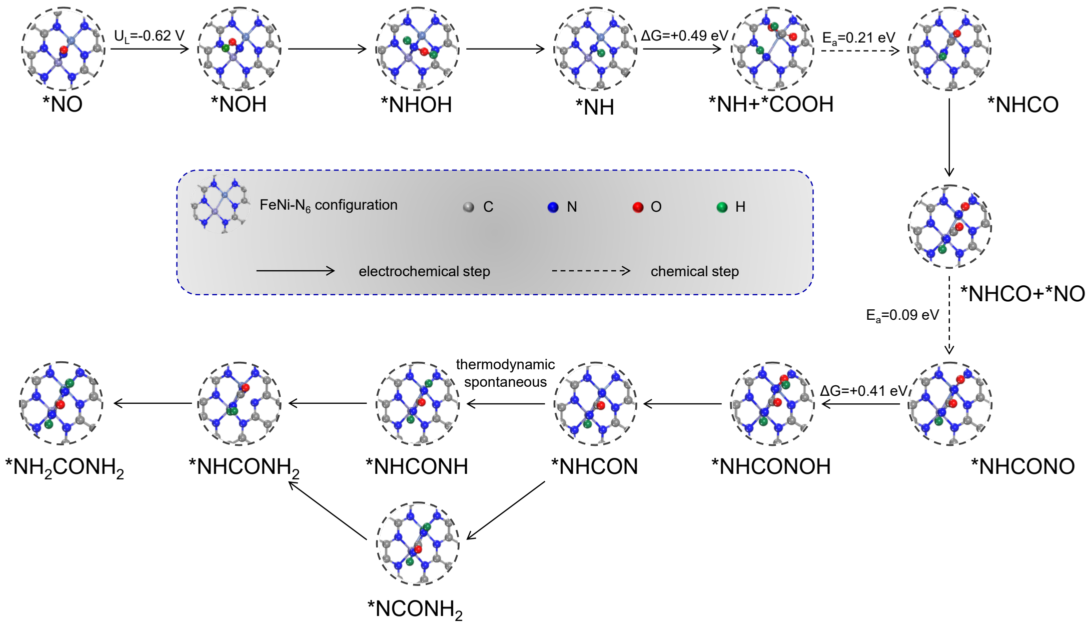

Supplementary Figure 31 | The structural modeling along the urea synthetic path over FeNi-N_6 configuration. The inset values are the Gibbs free energy changes and energy barriers, respectively.

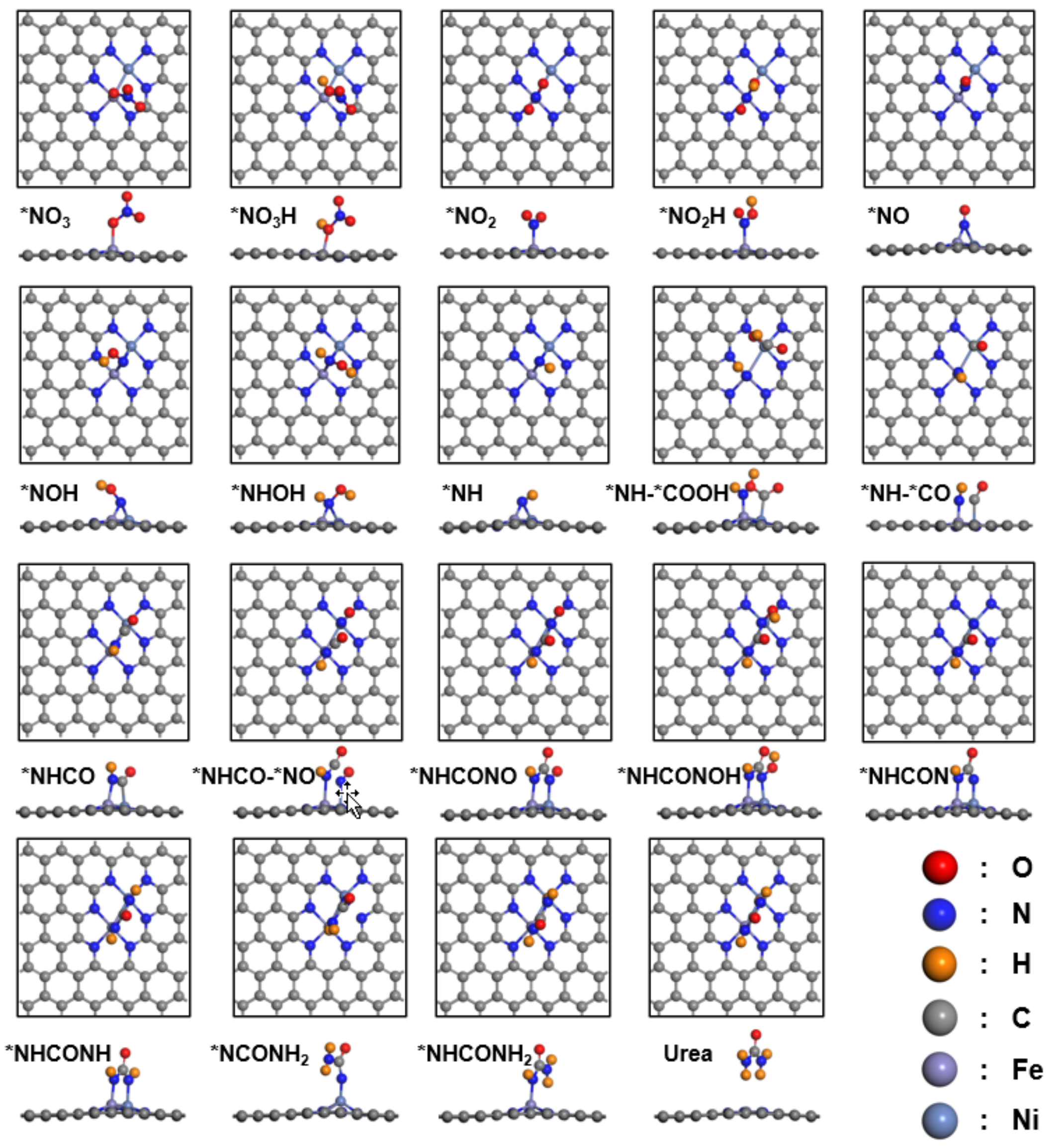

Supplementary Figure 32 | The top and side view of intermediates involved in urea formation on FeNi-N_6 slab. The red, gray, blue, purple, orange and cyan balls represent O, C, N, Fe, H and Ni atoms respectively.

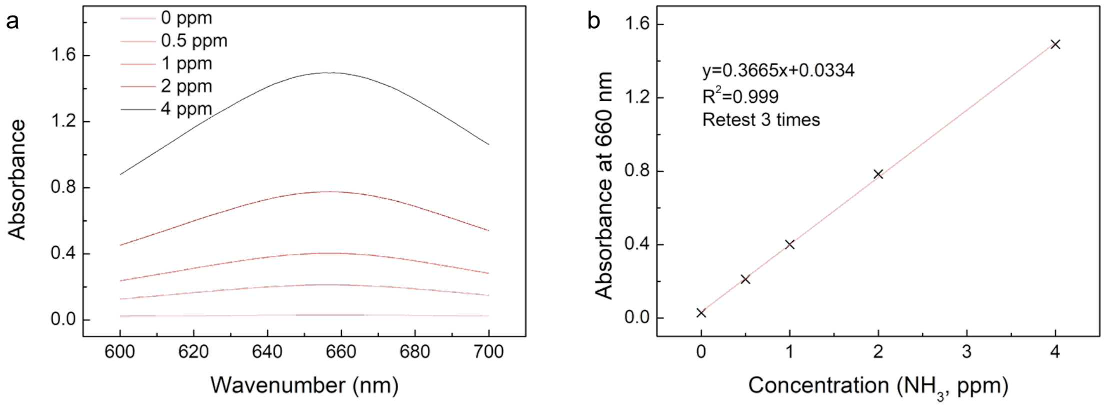

Supplementary Figure 33 | The quantification of ammonia by the indophenols blue method[4]. (a) The absorption curves of indophenols blue method with various concentrations of ammonia. (b) The calibration curve for ammonia quantification, the absorbance at 660 nm exhibits linear relationship with the concentration of ammonia ($y=0.3665x+0.0334$, $R^2=0.999$).

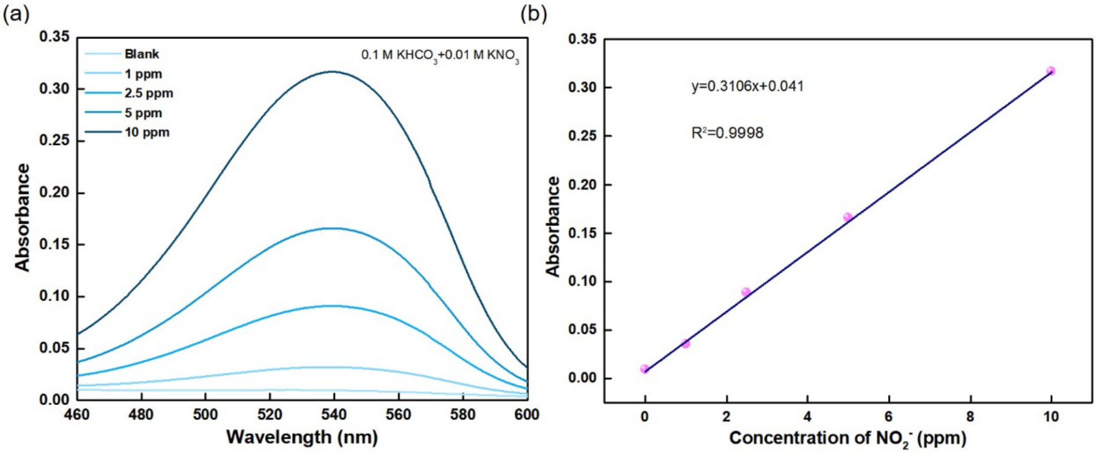

Supplementary Figure 34 | (a) A series of standard solutions with NO_2^- concentrations of 0 ppm, 1.0 ppm, 2.5 ppm, 5.0 ppm and 10.0 ppm respectively. (b) Calibration curve used for quantification of NO_2^- concentration. The absorbance at 540 nm was measured by UV-vis spectrophotometer, and the fitting curve shows good linear relation of absorbance with ammonia concentration (y=0.3106x+0.041, R^2=0.9998).

NO_2^- was determined using N-(1-naphthyl)-ethylenediamine dihydrochloride spectrophotometric method with some modification. Desailly, 0.5 g of sulfanilic acid was dissolved in the mixture of 90 mL of H_2O and 5 mL of acetic acid. Then, 5 mg of n-(1-naphthyl)-ethylenediamine dihydrochloride was added and the solution was filled to 100 mL to obtain chromogenic agent. 1 mL of the treated electrolyte was mixed with 4 mL of chromogenic agent and kept in dark for 15 min. The UV-vis absorption spectrum was then acquired at 540 nm.

Supplementary Table 1 | Fe and Ni K-edge EXAFS curve-fitting parameters for SACs and reference samples.

Samples	Path	N	R(Å)	σ^2($\times 10^{-3}$ Å^2)	ΔE_0	R-factor
**Fe-foil**	Fe-Fe	7.8±0.4	2.46±0.002	5.0±0.46	5.5±1.9	0.02
**Ni-foil**	Ni-Ni	12±0.5	2.47±0.002	5.0±0.62	6.4±1.3	0.0176
**B-FeNi-DASC**	Fe-Ni	5.8±0.4	2.49±0.001	8.0±0.34	6.2±1.2	0.016
**I-FeNi-DASC**	Fe-N	3.8±0.7	2.04±0.001	8.0±0.56	5.3±1.3	0.04
**I-FeNi-DASC**	Ni-N	3.8±0.4	1.99±0.002	7.0±0.75	5.3±2.0	0.04
**Fe-SAC**	Fe-N	3.9±0.2	2.09±0.001	6.0±0.63	4.2±2.2	0.033
**Ni-SAC**	Ni-N	3.8±0.6	2.10±0.001	8.1±0.49	6.9±2.5	0.04

Note: *N* is coordination number, *R* is the distance between absorber and backscatter atoms, σ^2 is Debye-Waller Factor, E_{shift} is inner potential correction; *R*-factor indicates the goodness of the fit.

Supplementary Table 2 | The quality contents (wt%) of Fe, Ni, C, O and N elements in the catalysts.

Electrocatalysts	Fe	Ni	C	O	N
**Fe-SAC**	1.792	–	89.98	4.088	4.14
**Ni-SAC**	–	1.566	90.83	3.284	4.32
**I-FeNi-DASC**	0.992	0.879	90.12	3.619	4.39
**B-FeNi-DASC**	0.991	0.896	89.84	3.863	4.41

Supplementary References

1. Zhang, X. et al. N species tuning strategy in N, S co-doped graphene nanosheets for electrocatalytic activity and selectivity of oxygen redox reactions. *Chem. Eng. J.* **431**, 133216 (2021).
2. Liu, J. et al. High performance platinum single atom electrocatalyst for oxygen reduction reaction. *Nat. Commun.* **8**, 1-10 (2017).
3. Xu, Y. et al. Highly nitrogen doped carbon nanofibers with superior rate capability and cyclability for potassium ion batteries. *Nat. Commun.* **9**, 1-11 (2018).
4. Chen, C. et al. B-N Pairs Enriched Defective Carbon Nanosheets for Ammonia Synthesis with High Efficiency. *Small* **15**, 1805029 (2019).
